# 29 Efficacy of a Novel LAM Femoral Cutaneous Block Technique for Acute Donor Site Pain

**DOI:** 10.1093/jbcr/irac012.032

**Published:** 2022-03-23

**Authors:** David M Hill, Sai R Velamuri, Jay P Desai, Austin Ly, Eric Ly, Jerry Jones

**Affiliations:** Firefighters' Burn Center, Memphis, Tennessee; Firefighter's Burn Center, Memphis, Tennessee; St. Louis University Health, St. Louis, Missouri; University of Tennessee Health Science Center, Memphis, Tennessee; University of Tennessee Health Science Center, Memphis, Tennessee; University of Tennessee Health Science Center, Memphis, Tennessee

## Abstract

**Introduction:**

Patients with severe burn injuries often require split thickness skin grafting to expedite would healing with the thigh being a common donor site. Uncontrolled pain is associated with increased opioid consumption, longer lengths of stay, and delay in functional recovery. Regional nerve blocks are increasing in popularity although supportive literature is limited, and techniques vary. Recently, we presented a case demonstrating a novel LAM (lateral, anterior, medial) femoral cutaneous block technique. The purpose of the case series is to assess the safety, feasibility, and clinical efficacy in a larger cohort.

**Methods:**

The study was a dual IRB approved, observational case series from a single verified burn center. The electronic health record was retrospectively reviewed for patients admitted between June 2018 to June 2020 who had the LAM block placed for donor site pain by the acute pain service (APS) team. Patient demographics, and data pertinent to the LAM regional anesthesia block were collected (eg opioid usage before and after the block, pain, and physical therapy outcomes). The data were tabulated and analyzed using Microsoft Excel© and SPSS version 27.0. Descriptive statistics were utilized to describe the patient demographics and LAM block feasibility & safety. Morphine Milligram Equivalent (MME) were statistically compared utilizing repeated measures ANOVA on ranks and graphically presented.

**Results:**

Twenty-four patients had total of 27 blocks placed, where 3 patients received the LAM block on two separate occasions. One patient had bilateral LAM blocks placed and was included as the single instance for the analysis. The majority were Caucasian males, but mechanism of injury varied. Two-thirds had a neurologic or psychiatric history. Seventy-one percent used tobacco and a quarter had a history of polysubstance abuse. Median area of the donor site was 360 cm2 (207, 1140). Median day from admission to LAM was 8 (3, 12) with a median duration of 4 (3, 5) days. Median day until first ambulation after LAM was 3 (1,3). Eighty percent reported a decreased temperature sensation at the donor site. Pain was adequately controlled, and there were no adverse events or quadricep weakness noted. There was a significant reduction in MME after block placement (Figure 1).

**Conclusions:**

Regional nerve blocks offer an advantageous means of analgesia, while reducing potential adverse events associated with opioids. While the novel LAM technique reduced some sensation, early ambulation was not inhibited and patients able to participate with rehabilitation therapy.

